# Dietary Supplements—For Whom? The Current State of Knowledge about the Health Effects of Selected Supplement Use

**DOI:** 10.3390/ijerph18178897

**Published:** 2021-08-24

**Authors:** Regina Ewa Wierzejska

**Affiliations:** Department of Nutrition and Nutritional Value of Food, National Institute of Public Health NIH—National Research Institute, Chocimska St. 24, 00-791 Warsaw, Poland; rwierzejska@pzh.gov.pl

**Keywords:** dietary supplements, legal regulations, health, safety

## Abstract

Dietary supplements are products containing nutrients sold in various medicinal forms, and their widespread use may stem from the conviction that a preparation that looks like a drug must have therapeutic properties. The aim of this scoping review is to present what is known about the effects of using selected dietary supplements in the context of chronic diseases, as well as the risks associated with their use. The literature shows that the taking of vitamin and mineral supplements by healthy people neither lowers their risk of cardiovascular diseases nor prevents the development of malignancies. Many scientific societies recognize that omega-3 fatty acids lower blood triglycerides, but whether taking them prevents heart disease is less clear-cut. Taking weight loss supplements is not an effective method of fighting obesity. Often, some supplements are increasingly sold illegally, which is then also associated with the higher risk that they may be adulterated with banned substances, thus making them even more dangerous and potentially life-threatening. Supplements are necessary in cases of nutrient deficiency; however, even though prescription is not required, their use should be recommended and monitored by a physician.

## 1. Introduction

In accordance with the European Union law, as well as the United States law, dietary supplements mean products that are concentrated sources of vitamins, minerals, or other substances with a nutritional or physiological effect (e.g., amino acids, essential fatty acids, probiotics, plants, and herbal extracts) intended to supplement the regular diet. Dietary supplements are produced in the form of capsules, tablets, pills, and other similar forms, designed to be taken in measured small unit quantities [1,2]. Dietary supplements, despite their route of administration and drug-like appearance, have been classified as foodstuffs and not medicines. Thus, in formal terms, supplement users are consumers rather than patients, but the question remains whether it is sick or healthy individuals who should be the primary users. The market for dietary supplements continues to expand at a rapid pace, and manufacturers develop products for health problems affecting almost all organs of the body, as well as for non-existing conditions, e.g., supplements for bladder elasticity. Additionally, a growing awareness among the public about the role of nutrition in the maintenance of health, together with widespread beliefs about today’s food being poor in vitamins (e.g., due to impoverished soil), are among the reasons for dietary supplement use becoming common practice or even the norm.

Skyrocketing sales of supplements in times of the widespread accessibility and overconsumption of a wide range of foods may be described as the “supplement use phenomenon”. In the US, over 50% of adults declare supplement use, and in some studies almost 40% had taken dietary supplements during the previous 30 days when they were questioned [3,4]. In Poland, supplement use has been reported by 30–78% of adolescents and adults [5,6,7] and by approximately 40% of children [8]. Only one-quarter of supplement users take supplements recommended by their physician, meaning the overwhelming majority use dietary supplements of their own accord [9]. The primary reasons for supplement use in the United States are for overall health and wellness and to fill nutrient gaps in the diet [10], while in Poland, among the elderly it is a need to boost the immune system, and among younger people, use is related to improving the health and condition of skin and hair [7]. Moreover, more than 40% of Poles surveyed believe that taking vitamin and mineral supplements prevents diseases in healthy people, and almost 70% claim that the use of antioxidants prevents the development of cancers [11,12].

Due to the scale of supplement use, it is prudent to investigate whether and to what extent nutrient supplementation has beneficial effects and whether it lowers the risk of developing modern diseases. The aim of this scoping review is to present the state of knowledge about the effects of using selected dietary supplements in the context of the risk of developing non-communicable diseases typical of industrial societies, i.e., cardiovascular diseases, malignancies, and obesity, as well as supplements’ possible treatment-supporting effects. Additionally, supplement safety, including their contamination with banned substances, is discussed.

## 2. Materials and Methods

In view of the vast quantities of supplements of different compositions and purposes, the author chose to review published scientific research on the most frequently taken dietary supplements, both in Poland and worldwide, which are primarily vitamins and minerals, followed by omega-3 fatty acids [5,7,13,14]. In the case of dietary supplements for weight loss, the most used ingredients were considered, i.e., chromium, chitosan, green tea extract, bitter orange, and *Garcinia cambogia* [15,16].

Articles written in English were searched in PubMed and Medline using keywords (such as “dietary supplement”, “food supplement”, “vitamin–mineral preparations”, “vitamins”, “minerals”, “omega-3 fatty acids”, “weight loss supplements”, “chromium”, “chitosan”, “green tea extract”, “bitter orange” (*Citrus aurantium*), and “*Garcinia cambogia*” accompanied by the names of specific diseases (cardiovascular diseases, cancers, obesity) or the terms “safety” or “adulteration”. This article mainly covers publications from the last decade, including randomized placebo-controlled trials, meta-analyses of such trials, and other original studies, as well as position statements by scientific societies. Due to the breadth of the issue, the literature review was limited to those studies involving adults, excluding studies on pregnant women.

## 3. Results

### 3.1. Dietary Supplements and Cardiovascular Diseases

Fatty saltwater fish, fruit, and vegetables are the most common dietary components contributing to cardiovascular disease prevention. The literature offers conclusive evidence that a high intake of omega-3 fatty acids from fish and seafood lowers the risk of cardiovascular disease incidence and mortality [17,18] and that disease incidence is inversely correlated with fruit and vegetable consumption [19,20].

#### 3.1.1. Polyunsaturated Omega-3 Fatty Acids

The beneficial effect of omega-3 fatty acids, especially eicosapentaenoic acid (EPA), and docosahexaenoic acid (DHA), consists in their ability to reduce triglyceride concentration and to lower arterial pressure, as well as to inhibit inflammatory process and blood cell aggregation [17,21]. The well-documented importance of fish in the prevention of cardiovascular diseases resulted in a speedy market reaction and release of fish oil nutraceuticals. The question remains whether taking marine omega-3 fatty acid preparations is as beneficial as consuming marine omega-3 fatty acid foods and whether they may become an alternative product for people who do not consume fish.

Regarding the primary prevention of cardiovascular disease, the results of two large, randomized trials from recent years should be cited first. One of them is the British ASCEND study that was conducted with a cohort of 15,480 patients (age ≥ 40 years) with diabetes showing no evidence of atherosclerotic cardiovascular disease, who were administered either 840 mg of marine omega-3 fatty acids or a placebo for an average of 7.4 years. This study showed no significant difference in the primary endpoints (i.e., non-fatal myocardial infarction, stroke, transient ischemic attack, or vascular death) or the secondary endpoints (first serious vascular event or any arterial revascularization) between the study and control groups. The authors concluded that the results of their study did not show that omega-3 fatty acids supplementation prevents vascular events [22]. In an American trial, the VITAL study, conducted with a group of 25,871 healthy individuals aged ≥ 50 years, results revealed that consumption of 1 g of fish oil/day (840 mg EPA and DHA) for a median of 5.3 years did not lower the risk of myocardial infarction, stroke, or overall cardiovascular mortality in the study group compared with the placebo group [23].

Studies have also assessed omega-3 fatty acid supplementation in relation to secondary prevention. The REDUCE-IT Clinical Trial showed that administration of highly purified EPA ethyl ester (icosapent ethyl) at 4 g (2 g, twice daily) for a median of 4.9 years to patients with atherosclerotic cardiovascular disease or diabetes, who have elevated triglycerides and are treated with statins, reduced the risk of cardiovascular death, non-fatal myocardial infarction, non-fatal stroke, coronary revascularization, or unstable angina by 25%, compared with placebo, and reduced the risk of the secondary endpoint (cardiovascular death, non-fatal myocardial infarction, or non-fatal stroke) by 26%. The authors emphasized that the benefits demonstrated in their study should not be transferred to all omega-3 preparations, especially dietary supplements, due to the differences between the composition of supplements compared with the preparation used in their study [24]. The literature also shows that 4 g of icosapent ethyl improves the lipid profile in patients with statin intolerance [25]. Many other experts also emphasize that omega-3 fatty acids (of proven quality) may be particularly beneficial for statin-intolerant patients with obesity, diabetes, or metabolic syndrome in whom elevated LDL cholesterol is accompanied by high levels of triglycerides [26].

However, the STRENGTH randomized clinical trial of 2020 did not provide optimistic results. In that study, administration of 4 g of an omega-3 preparation with increased bioavailability (carboxylic acid formulation of EPA and DHA) to high cardiovascular risk patients treated with statins for more than 3 years proved of no benefit in terms of major cardiovascular events, and this finding resulted in early termination of the study [27]. As experts point out, such divergent results of single studies may result from different proportions of individual fatty acids in the preparations, the doses of the preparations, the length of the study, or the varying initial concentrations of omega-3 in the blood of the patients [28].

To obtain clearer data in such situations, conclusions from meta-analyses of randomized controlled trials are typically used, and though these have been quite numerous in the past 10 years, their results do not provide sufficient evidence to form an unambiguous conclusion. The findings of three 2012 meta-analyses showed that omega-3 fatty acid supplementation did not lower the risk of myocardial infarction, stroke, cerebrovascular events, cardiovascular mortality [29,30], or cardiovascular incidents in patients with existing cardiovascular diseases [31]. A meta-analysis published one year later revealed that supplementation with omega-3 fatty acids reduced the risk of myocardial infarction by 25% and the risk of cardiac death by 32% in patients with cardiovascular diseases [32], while a meta-analysis published in 2014 reported a 12% reduction in death from cardiac causes in patients with coronary heart disease who took omega-3 fatty acids [33]. In turn, a 2018 meta-analysis of randomized studies of omega-3 fatty acids taken, showed no effects on non-fatal myocardial infarction, coronary heart disease events, or major vascular events [34], a finding that is consistent with another 2018 meta-analysis that also showed no relationship between omega-3 fatty acids consumption and lowered risk of cardiovascular disorders [35]. In contrast, the results of one of two 2019 meta-analyses of randomized clinical trials showed that taking omega-3 fatty acids reduced the risk of major vascular events by 5%, non-fatal myocardial infarction by 11%, and death by coronary heart disease by 9% [36]. The second 2019 meta-analysis of the studies, from which the REDUCE-IT study was excluded because of the comparatively high level of its omega-3 dose, showed that omega-3 supplementation was associated with lower risk of myocardial infarction by 8%, total coronary heart disease by 5%, coronary heart disease death by 8%, and cardiovascular death by 7% [37]. Further evidence of the beneficial effect of omega-3 fatty acids in reducing cardiovascular risk is also found in the results of two meta-analyses from 2020 [38,39], although there was also an associated higher risk of bleeding events and atrial fibrillation events in some instances [39].

Against the background of the current state of scientific knowledge, what are the positions of the scientific societies responsible for recommendations to the medical community?

The International Lipid Expert Panel (ILEP) stated in 2017 that omega-3 fatty acids are proven to lower triglycerides (scientific evidence: class I, level A) [40,41] and added to this view in 2020 by indicating the need for EPA and DHA supplementation in heart failure, especially in patients after myocardial infarction [42]. According to the ILEP, omega-3 fatty acids in doses of 1–4 g daily reduce triglyceride levels by 18–25% [40]. The European Society of Cardiology (ESC) and the European Atherosclerosis Society (EAS) both state that doses of 2–3 g reduce triglyceride levels by about 30% [43]. The European Society of Cardiology and other societies on cardiovascular disease prevention in clinical practice, as of 2019, did not recommend omega-3 fatty acid supplementation for cardiovascular diseases prevention due to the lack of reliable proof of its beneficial effects [20]. Meanwhile, the 2019 American College of Cardiology/American Heart Association guideline on the primary prevention of cardiovascular disease makes no mention of omega-3 fatty acids [44], which should be understood as a lack of support for their use. The American Heart Association stated in 2017 that supplementation of omega-3 fatty acids would be beneficial in patients with coronary heart disease (CHD), including those after heart attack, as it may lower the risk of CHD-related mortality by 10% [45].

In conclusion, research findings on the impact of omega-3 fatty acid supplementation on the risks or benefits in relation to the treatment of cardiovascular diseases vary significantly. However, there are many indications that taking omega-3 fatty acid preparations by healthy people do not have such beneficial effects as eating the same fats in fish. Therefore, consumption of omega-3 capsules does not offer an easy way to improve nutrition and, apparently, cannot serve as a substitute for fish and seafoods in the diet. Importantly, recent years have brought a growing number of reports about low-quality fish oil supplements [46], which according to various authors are nowhere near as good as preparations registered as medicines [47]. In New Zealand, as many as 83% of supplements containing fish oils were shown to exceed permissible levels of peroxides, which are an indicator of fat deterioration [48]; similarly, in the US, oxidized fatty acids, cholesterol, and toxins were found in many omega-3 supplements [49]. In Poland, there is a wide range of supplement products on the market containing omega-3 fatty acids and, as is the case throughout the European Union, there is no legal requirement for standardization of such preparations regarding individual fatty acids content [21]. Currently, however, there are no data on the contamination of such preparations in Poland.

#### 3.1.2. Vitamins and Minerals

Vitamins and minerals are essential substances that our bodies need for optimal functioning. As there is a considerable selection of vitamin and mineral supplements, it seems prudent to consider whether they bring any benefits in terms of cardiovascular disease prevention. As far as arterial hypertension is concerned, a prospective study by Rautiainen et al. [50] found no relationship between vitamin supplementation and the risk of developing hypertension in healthy women. Wang et al. [51], who investigated obese women with increased cardiovascular disease risk in a randomized study, reported that a 26-week supplementation with a vitamin–mineral preparation significantly lowered blood pressure compared with the placebo given to the control group.

The authors of the 2018 meta-analysis of randomized studies concluded that vitamin–mineral supplementation does not lower the risk of developing hypertension but, at the same time, suggested that such preparations may have a beneficial effect in subjects with hypertension [52]. The benefits of potassium supplementation have been extensively documented. Experts believe that potassium supplementation in patients with hypertension supports treatment and may be recommended [53,54].

As for other endpoints of studies on vitamins and minerals intake and cardiovascular diseases, Park et al. [55] conducted a study in over 180,000 people in the US and found that multivitamin preparations do not inversely correlate with cardiovascular mortality. Additionally, a randomized study in the US with more than 14,000 physicians reported no positive effects of supplementation, either with single-preparation vitamin C, E, and beta-carotene or multivitamin preparation, with regard to cardiovascular incidents in either healthy subjects or individuals with cardiovascular disease [56]. Randomized trials also do not reveal the benefits of B vitamins (folic acid, vitamins B6, and B12) supplementation in patients with pre-existing cardiovascular disease. Equally, in the study by Albert et al. and Galan et al., no significant effect on the risk of major cardiovascular events was found [57,58]. However, it should be mentioned that optimistic results were obtained in a Chinese study of taking enalapril (a drug for treating high blood pressure) in combination with folic acid. In this large randomized study, conducted among adults with hypertension without a history of stroke or myocardial infarction, the combined use of enalapril and folic acid (0.8 mg/day), compared with enalapril alone, reduced the risk of the first stroke by 21% and the risk of composite cardiovascular events by 20% [59].

A meta-analysis of clinical trials and prospective cohort studies reported no relationship between vitamin–mineral supplementation and the incidence of stroke and the risk of cardiovascular mortality in the general population [60]. An umbrella review of 2019 [61] also found no effect of vitamin preparations on the development of cardiovascular diseases and cardiovascular mortality. For years, the US Preventive Services Task Force panel of experts has not been recommending vitamin–mineral supplementation for cardiovascular disease prevention in people without confirmed insufficiency [62,63].

#### 3.1.3. Antioxidants

Although no universal definition of antioxidants exists in the literature, it is believed that they are compounds that donate one of their electrons or hydrogen to free radicals, stopping their chain reaction [64]. Some vitamins and minerals (vitamins A, C, E, beta-carotene, selenium, and zinc) are classified as antioxidants and are a subcategory of dietary supplements [65]. Antioxidants prevent the negative effects of free radicals, inhibit the oxidative process, and reduce inflammation in the body. The deficiency of these nutrients in the body may increase the risk of developing a variety of conditions, including cardiovascular diseases [52,66]. Antioxidant supplementation has been extensively researched, but the findings remain inconclusive, especially in the case of vitamin E [13,67,68].

In a study by Lee et al. [69], daily supplementation with 600 IU of vitamin E by healthy women for 10 years did not alter the incidence of myocardial infarction and stroke as compared with the study’s control group taking placebos, but it did lower the risk of cardiovascular mortality by 24%. Lonn et al., [70] not only found that vitamin E supplementation (400 IU/day for a median of 7 years) failed to reduce the risk of cardiovascular incidence in affected individuals but also found that it increased the risk of heart failure by 13%. The results of a recently published Mendelian randomization study indicated that genetically conditioned higher blood concentrations of vitamin E elevated the concentrations of LDL cholesterol and triglycerides, decreased HDL concentration, and increased the risk of coronary artery disease [71].

The results of several meta-analyses of randomized studies published over the years also remain inconclusive. In a 2003 meta-analysis of multiple studies, vitamin E (50–800 IU) administered for 1.4–12 years was shown to have no beneficial effect on cardiovascular mortality rates, and its routine use was not recommended [72]. A meta-analysis published in 2015 concluded that vitamin E supplementation reduces the risk of cardiac infarction, but the effect is negated when it is administered together with other antioxidants [73]. Yet another meta-analysis from 2017 reported beneficial effects of single-preparation vitamin E—namely, a 12% reduction of the risk of cardiovascular mortality. However, the authors found no justification for administration of other antioxidants, i.e., vitamin C, selenium, and zinc [13]. The International Lipid Expert Panel believes that current research results do not show any benefits of vitamin E supplementation in the prevention or treatment of heart failure, and they draw a similar conclusion for vitamin C [40].

Findings regarding beta-carotene are less optimistic. According to a meta-analysis of randomized studies, beta-carotene at a dose of 15–50 mg for 1.4–12 years has led to a slight but statistically significant increase in cardiovascular mortality [72]. The US Preventive Services Task Force panel of experts also opposes the use of both beta-carotene and vitamin E in cardiovascular disease prevention [74]. The most radical position on antioxidant supplementation has been taken by the authors of the “Enough is enough: Stop wasting money on vitamin and mineral supplements”, who claim that a sufficient number of studies have demonstrated the lack of benefits of vitamin–mineral supplementation to warrant ceasing any further research on their effectiveness [75].

In summary, based on the available literature, it seems safe to conclude that supplementation with vitamin–mineral preparations whose intake exceeds the needs of the body is not a recommended method of cardiovascular disease prevention. Table 1 summarizes the studies included in this paper, showing relationships between dietary supplement use and cardiovascular health.

### 3.2. Dietary Supplements and Malignancy

#### 3.2.1. Cancer Prevention

According to the literature, the effects of vitamin and mineral consumption on the risk of developing cancer has a U-shaped distribution (the dose–response curve). Optimal intake of these components (the range that corresponds with the base of the curve) is associated with lower risk of the disease, whereas both insufficiency and excessive consumption may promote carcinogenesis [9,76].

In light of the growing popularity of the so-called “Western diet”, it seems prudent to ask whether vitamin and mineral supplementation may in fact reduce the development of cancer and whether it brings any additional benefits in people with healthy diets. A randomized study of vitamin C (500 mg/day) and E (400 IU every other day) supplementation and cancer incidence in men, by Gaziano et al., [77] found no differences between the study group and the placebo control group; similarly, Lee et al. [69] demonstrated no benefits of vitamin E supplementation (600 IU every other day) in women. The results of a multi-center Selenium and Vitamin E Cancer Prevention Trial are even less favorable, finding that vitamin E supplementation (400 IU/day) increased the risk of prostate cancer by 17% [78]. Additionally, in a long-term prospective study by Park et al., no relationship was found between multivitamin supplementation and cancer development [55].

All hope that health improvements derive from vitamin supplementation was abandoned after the results of studies on beta-carotene were published. In fact, those studies may even be considered as an argument against supplementation. There is an accumulating body of evidence that suggests that beta-carotene, a typical antioxidant, may in fact exert a pro-oxidative effect in smokers [79]. CARET, one of the first randomized trials conducted in the 1980s, demonstrated that administration of 30 mg of beta-carotene and 25,000 UI of retinol per day for an average of 4 years resulted in a 28% increased risk of lung cancer and a 17% higher risk of mortality [80]. These unexpected results led to the study being prematurely terminated. Another randomized study, conducted with male smokers from Finland between 1985 and 1993, included a similar conclusion. Supplementation with beta-carotene at a dose of 20 mg/day for an average of 6 years increased the risk of lung cancer by 16% [81], and further analysis showed that the increased risk did not correlate with nicotine and tar contents in the cigarettes [82]. A Japanese study on vitamin A intake in the diet also generated unfavorable results. Higher consumption of vitamin A was associated with a 26% greater risk of lung cancer [83]. Based on the meta-analyses of high-quality randomized trials, it seems that antioxidant administration (vitamin A, C, E, selenium, and zinc combined) in healthy individuals does not lower the risk of developing lung cancer. Vitamin C supplementation increased the risk of malignancy in women by 84%, and similarly, vitamin A increased the risk in smokers and people with history of asbestos exposure by 10% [84]. Studies have also shown that antioxidant supplementation produces no benefits in the prevention of bladder [79] or colorectal cancers [85].

According to some experts, antioxidant supplementation in cancer prevention may only be beneficial in healthy people with vitamin insufficiency. High doses of antioxidants may even be harmful to people in the subclinical phases of the disease [76,86]. Selenium is a good example of the thin line between beneficial and detrimental doses when the relationship between antioxidant consumption and the risks of developing cancer are concerned. In people with low blood concentrations of selenium, its supplementation lowers cancer risk, but in individuals with high blood concentrations of selenium, supplementation increases the risk of developing lung cancer [87,88].

Vitamin D supplementation has also caused a lot of interest in recent years in relation to cancer prevention because of the vitamin’s anti-inflammatory and immunomodulatory properties and the presence of Vitamin D Receptor (VDR) in most human organs. However, meta-analysis of randomized controlled trials has not confirmed that vitamin D intake reduces the incidence of cancer [89,90,91].

Summarizing the current state of knowledge about vitamin and mineral supplementation in cancer prevention, it is important to emphasize that the expert panel of the World Cancer Research Fund opposes the use of such preparations [92], as does the American Cancer Society, which, in addition, has concluded that if, despite the absence of benefits, one chooses to take vitamin–mineral supplements, doses exceeding 100% of the daily demand should not be used [76]. According to the U.S. Preventive Services Task Force panel of experts, we lack sufficient data to determine the role of vitamin preparations in cancer prevention, except for beta-carotene and vitamin E, whose supplementation is universally discouraged [74].

#### 3.2.2. Cancer Therapy

Supplement use among cancer patients is common and typically unsupervised by a physician. Alternative medicine therapies, including “word-of-mouth” dietary supplement recommendations, are believed to be safe and complementary to the therapeutic process [74,88]. A study by Mandecka et al. [66] demonstrated that over 46% of cancer patients used dietary supplements, mostly antioxidants, although the patients’ mean intake of vitamin A, C, and E from food ranged between 200% and 300% of the daily requirements. According to the literature, 64–81% of cancer patients take vitamins or minerals, and 26–77% use multivitamin preparations [93,94]. Presumably, three-quarters of the physicians are not aware of what the patients take of their own accord, following the rule “patient keeps quiet, doctor asks no questions”. The case of shark cartilage is an interesting example of the difference between popular perception and proven efficacy, as it was extremely popular in the 1990s, although it was utterly ineffective as a dietary supplement allegedly helpful in fighting cancer [95].

Vitamin C, due to its antioxidative action and positive effect on the immune system, has been immensely popular among cancer patients. However, randomized studies showed no indication that supplementation may provide favorable results [96]. In the case of the impact of vitamin D supplementation, some meta-analyses of randomized controlled trials indicate that vitamin D intake reduces the risk of cancer mortality by 12–13% [90,91]. However, some authors note that due to the low quality of the research, this finding should be approached with caution [91] and yet others do not confirm any relationship [89]. Decisions about possible vitamin D supplementation in cancer patients should consider the current and target 25 (OH) D concentrations in the patient’s blood [90].

In fact, a considerable number of the experts believe that cancer patients should refrain from any supplementation, especially during chemotherapy and radiation therapy [66,97,98]. According to Ambrosone et al. [99], antioxidant supplementation in breast cancer patients undergoing chemotherapy lowered the chance of survival. The American Institute for Cancer Research warned that patients undergoing chemotherapy or radiation should not ingest high doses of vitamins and minerals [100]. Vitamin–mineral preparations are necessary in cases of patient malnutrition. According to the European Society for Clinical Nutrition and Metabolism (ESPEN), cancer patients whose diet provides them with less than 60% of the required caloric content over the course of at least 1 week, should use vitamin–mineral supplements in adequate doses so that they avoid nutritional deficiencies. However, supplementation should be prescribed and monitored by a physician [87,88]. Importantly, more than half of cancer patients declare they received no information whatsoever regarding vitamin and mineral supplementation during therapy [101].

Herbal supplements, which are commonly perceived by patients as completely safe due to their natural origin, are particularly dangerous [102,103,104]. The risk is associated, among others, with the higher content of active substances in the supplements than occur in the natural herbal resources, which results from the use of concentrated extracts and sometimes additional synthetic analogues [105,106]. Additionally, recent years have witnessed the appearance of numerous preparations that include plants that have never previously been used in Western medicine. Their mechanisms of action have not been sufficiently investigated and described, while the labels usually fail to include information about the contraindications, which does not mean they do not exist. Herbal components, especially herbal mixes, may have a negative effect on the drug mechanisms of action, both by accelerating excretion from the body or by producing dangerously high concentrations in the blood [88,104].

The abovementioned data indicate that reasonable supplementation, tailor-made for the cancer patient and supervised by a physician, may in fact bring benefits and constitute a vital element of the cancer treatment. Unsupervised self-administration of dietary supplements may cause serious complications during cancer therapy. Table 2 summarizes the studies included in the paper on the relationships between vitamin–mineral supplementation and cancer.

### 3.3. Dietary Supplements and Weight Loss

The number of people with excessive weight continues to rise, and fighting obesity has become one of the greatest challenges of contemporary medicine. A person wishing to lose weight needs to undertake several difficult life-changes and practice them consistently (diet, physical activity, addiction-free). Meanwhile dietary supplements are presented as a compelling alternative to traditional methods for combatting obesity. Wróbel-Harmas et al. [107] demonstrated that weight loss supplements are the most frequently sought dietary supplements on the Internet, followed by preparations for muscle building and sexual potency. In the US, more than 30% of people with overweight and obesity believe supplements to be an effective method for losing weight [108], while in Poland these supplements are used by as many as 40–50% of young women, regardless of their weight [15].

Weight loss supplements are usually multi-ingredient preparations, with over 4000 individual substances used in the production process. The average weight loss supplement available in Western markets is estimated to include 10 different ingredients [109]. The more complex the recipe, the harder it is to determine its effects on the body. The most popular ingredients include chromium and chitosan, as well as green tea, *Garcinia cambogia*, and bitter orange (*Citrus aurantium*) extracts [15,16]. Over the years, no studies have shown that the use of either single- or multi-ingredient preparations of those substances promotes weight reduction.

A 2013 meta-analysis of randomized studies found that chromium supplementation resulted in only 0.5 kg additional weight reduction in subjects with overweight and obesity, as compared with those taking a placebo [110], and a comparable result (mean: 0.75 kg) was achieved in a similar 2019 meta-analysis [111]. In addition, the literature indicates that chromium supplementation may be associated with several side effects: emesis, nettle-rash, and dizziness [112]. Claims about the benefits of chitosan, which allegedly binds to fats in the digestive tract and inhibits fat absorption, are also exaggerated [113,114]. A 2018 meta-analysis of randomized trials revealed that chitosan supplementation for approximately 17 weeks led to a slight decrease in body weight (mean: 1 kg) [113], and a similar result was found in a 2020 meta-analysis of similar studies, which reported a 0.89 kg greater weight loss than those taking a placebo [115]. The effectiveness of green tea extract is also questionable. In light of some data, green tea extract consumed for 12 weeks did not reinforce the weight loss process [116]; in light of other data, consumption of the extract for up to 14 weeks by obese people causes a greater decrease in body weight (by 1.8 kg) and BMI (by 0.65 kg/m^2^) than occurring in those taking a placebo [117]. At the same time, a growing number of studies have shown there is a significant risk linked with green tea extract, and we discuss this in more detail in the Safety of Supplement Use section of the manuscript. *Garcinia cambogia*, which is a tropical plant whose fruit includes large quantities of hydroxycitric acid, which inhibits appetite and suppresses fatty acid synthesis, has also been advertised by celebrities as a magical aid to losing weight [104,118,119]. The results of studies on the effectiveness of *Garcinia cambogia* in the weight loss process are far from satisfying. A meta-analysis of 2011 randomized controls demonstrated poor-to-borderline statistically significant effects on body weight loss (−0.88 kg) as compared with those on the placebo and demonstrated no effect on BMI. The authors pointed out the small sample size of the studies they analyzed and the fact that the studies were conducted over short periods of time [120]. Slightly better slimming effects of *Garcinia cambogia* supplements were shown by a 2020 meta-analysis of such studies, where taking such supplements for 8–12 weeks resulted in decreases in body weight by −1.34 kg, BMI by −0.99 kg/m^2^, percentage of fat mass by −0.42%, and waist circumference by −4.16 cm compared with the placebo group [121]. Overall, the effectiveness of *Garcinia cambogia* is believed to be under-researched, and long-term use is not recommended. Additionally, the plant is supposedly hepatotoxic [114,122]. Similarly, results to those above are found in the literature on bitter orange (*Citrus aurantium*), showing no conclusive proof of its effectiveness [107,122,123]. However, there are reports on its serious side effects (hypertension, chest pain, and tachycardia), especially among subjects with cardiovascular diseases, as well as indications that it may cause liver damage [124]. The effects of bitter orange alkaloids on liver enzyme activity may also produce negative effects on the efficacy of other medicines [112].

In summarizing current knowledge about weight loss supplements and their effectiveness, it is important to emphasize that none of the available supplements are recommended because their effectiveness is unproven and for safety reasons. Recipients who are exposed to the advertisements of such supplements should be aware that their alleged effectiveness has not been tested in clinical trials. Advertising slogans such as “excellent fat burner” have no grounds. Additionally, if there was a weight loss preparation that was proven to be effective, it would have been registered as a “medicine” and not as a “foodstuff”. The two key issues are that these products do not help people lose weight and they have serious side effects, which may be a threat to patient health. Therefore, consuming fat burners, especially those purchased online, has been compared by some authors to playing a game of “Russian roulette” [125]. If, despite the lack of evidence, patients wish to attempt to lose weight using dietary supplements, they should never purchase these products from unauthorized buyers or increase the recommended dose or use several products at the same time in the hope that it would accelerate their weight loss. Various slimming preparations (with different trade names) may contain the same ingredients and their concentration in the body may become dangerously elevated. Table 3 summarizes the studies analyzed in this article on the relationships between dietary supplement use and body weight.

### 3.4. Safety of Supplement Use

In Europe and the US, no documentation of safety of use is required before the introduction of a dietary supplement to the market, although each jurisdiction’s laws clearly state that producers are responsible for the safety of a product [124,126]. Some authors have stated that they believe that supplements are released onto the market to be tested by the end-users [127]. The Food and Drug Administration (FDA) may withdraw a product from the market only after it has been deemed unsafe [128,129]. Additionally, routine quality controls are not carried out to check the active substance content and label compliance [105,107], and yet half of some studies’ respondents claim that dietary supplements are sufficiently monitored, in the same way that over-the-counter drugs are [107,130].

Evidence of the unfavorable effects of supplement use continues to accumulate. According to the National Estimates of Emergency Department Visits, the most frequent complaints in the US concern herbal or complementary nutritional preparations, including weight loss products (66% and 26% of all interventions, respectively) [131]. As far as weight loss products are concerned, 13,000 complaints are registered annually, including 2000 hospitalizations [132]. The main side effects include cardiac symptoms (palpitations and chest pains), and the same symptoms have been observed in relation to the use of muscle building and sexual potency supplements [131]. The literature emphasizes that long-term effects of supplement use are difficult to predict and will only be known after they have been present on the market for some years [133]. The *Ephedra sinica* shrub is an interesting example of this issue, as it became a component of weight loss preparations in the early 1990s in the US and was withdrawn in 2004 due to the incidence of life-threatening cardiac side effects [112]. However, the use of the *Ephedra sinica* plant in foods, and consequently in dietary supplements, was not banned in the European Union until 2015 [134]. Ephedra herbal supplements accounted for over half of the complaints about adverse reactions to herbs lodged with the US National Estimates of Emergency Department Visits, yet these products constituted only 1% of total herbal product sales in the US at the time [112]. In 2004, the FDA received over 18,000 complaints and finally ruled that the product should be withdrawn [114]. Sibutramine (active ingredient in medicines) is yet another example of a substance used is weight loss preparations whose negative side effects were revealed only after people had been using it for some time. It was released on the market in the late 1990s as a component of an appetite-blocking drug but was withdrawn in 2010 due to a significant risk of cardiovascular disorders [135].

Since being official banned, both sibutramine and *Ephedra sinica* have become popular, illegal components of weight loss supplements. In China, significant quantities of sibutramine were detected in 27 out of 120 weight loss supplements [103]. Despite a number of components being listed on the label of one Chinese product, Meizitanc, when investigated in Poland, sibutramine was the only substance in this weight loss [136]. There is also an associated problem of additional ingredients that are also dangerous being included with the banned substances. For instance, the number of reports on laxatives, antidepressant, yohimbine, and even amphetamine and its derivatives being found in supplements continues to grow [105,106,127,137]. A US study revealed that 11 out of 21 supplements that contained the *Acacia rigidula* extract that were purchased on the Internet contained the isomer of amphetamine [138]. In South Korea, substances that bore a structural resemblance to amphetamine were found in 10 out of 110 weight loss supplements [139]. In Italy, 28% of the supplements purchased online contained sibutramine or substances only permitted in medicines or their analogs, which had not been tested for toxicology [135]. Apart from weight loss supplements, preparations for muscle building and sexual potency have also been found to be contaminated. International studies indicate that 12–58% of the supplements for physically active people contained substances that have previously been banned by the World Anti-Doping Agency [140].

Despite legal requirements, labels of dietary supplements cannot be treated as a source of reliable information about the products’ contents. In North America, where the brain health supplement market is developing rapidly, 10 out of 12 products contained non-declarable components, and 8 did not contain at least 1 of the ingredients listed on the label [141]. Overall, it is estimated that approximately 20% of the supplements contain at least one banned substance. Importantly, in view of the number of products available on the market, the published cases are merely the tip of the iceberg [105,106].

Lack of reliable data on supplement safety, contamination, and adulteration are the reasons why supplements may cause grave side effects. So far, most reports have focused on the green tea extract, which may have caused liver damage, including fatal damage, in more than 50 individuals. For example, Exolise—a weight loss supplement that contained green tea extract—was withdrawn from the market in Spain and France in 2003 [112,129]. Dexaprine was a multi-ingredient supplement with green tea extract that was withdrawn in 2014 in Holland, as even half a tablet resulted in emesis, anxiety, and tachycardia [106]. In Poland, anaphylactic reactions were observed in the case of a multi-ingredient preparation with green tea extract known as *Linea Detox* [133]. Supplements that contain *Garcinia cambogia* may also pose a threat to patient life. Two cases are Hydroxycut, which resulted in liver damage, heart arrhythmia, and death [118,124,132]; and Thermatrim, which was suspected of causing toxic leukoencephalopathy [142]. Kratom is yet another example of a dangerous dietary supplement for muscle building that was withdrawn in 2018 from the US market after it caused the death of a young man [128].

The rapid increase in the availability of dietary supplements aimed at improving cognitive function, including Alzheimer’s disease, is the reason for the FDA issuing an official statement in 2019 to inform the public that the agency did not investigate dietary supplements for safety and effectiveness and that these preparations may be dangerous, ineffective, and delay patients’ decisions to seek medical help [143]. In light of the fact that the disturbing reports we have identified above continue to surface, there is an urgent need for a radical change in the way supplements are introduced to the market [127,144,145], especially in changing perceptions of products from “safe until proven unsafe” to “unsafe until proven safe” [128]. There is a consensus among the experts that supplements require controlled tests to be performed by independent laboratories to check for active substance content and possible adulteration [105,106,107].

## 4. Discussion

Due to persistent media messaging about poor eating habits and that the typical “Western diet” is far from nutritional, many people are convinced that their daily diet does not meet the recommended levels for vitamins and minerals. However, the scientific literature shows that taking vitamins and minerals without medical justification does not lower the risks of cardiovascular disease and cancer and that sometimes it can even have a negative effect. It should also be borne in mind that disease is influenced by many factors, both genetic and environmental, and a beneficial change in one element of the diet may not have any overall effect. Popular positive expectations of supplements are also dampened by the fact that they are more often used by people who already eat better, lead a healthier lifestyle, are better educated, and have a greater health awareness [6,8,10]. Therefore, in such cases where the person already has a better nutritional status, including a lack of vitamin and mineral deficiencies, supplementation may not be of benefit, and long-term consumption of excessive quantities of these nutrients may, in fact, have detrimental side effects. The risk of such side effects increases when consumers take several dietary supplements at the same time (for example, “for immunity”, “for the heart”, or “for an efficient mind”), as they may contain the same vitamins and minerals as each other.

On the other hand, the beneficial effect of using certain nutrients (omega-3 fatty acids) in the treatment of lipid disorders, as demonstrated in studies, undermines the registration of preparations intended for this purpose as dietary supplements. Dietary supplements, by their definition, serve to supplement the usual diet with nutrients or components with a physiological effect, and this group of preparations does not include medicinal products [1,2,146]. Due to one of the basic rules that the use of preparations for therapeutic purposes should be prescribed and under the supervision of a physician, “therapeutic” doses of nutrients should not be used in food preparations. Moreover, Polish pharmaceutical law clearly emphasizes that in the case of a product that meets the criteria for classification both as a medicinal product and as a dietary supplement, the provisions of pharmaceutical legislation, rather than food regulations, should be applied [147]. Currently, the dose of active ingredients is an important criterion in the registration process of dietary supplements [148]. It is also worth remembering that labeling and advertising of dietary supplements can neither imply nor make claims about the product’s preventive and/or therapeutic properties, which is consistent with food law and the regulations governing all foodstuffs [149]. Therefore, dietary supplement packaging may not contain information such as “for cancer prevention” or “for reducing cholesterol”. However, although such requirements have long been in food legislation, the practice of portraying dietary supplements as products that quickly alleviate the symptoms of numerous health conditions, both by labelling and advertising methods, is relatively common [143,150,151,152]. Unsurprisingly, the public often finds it difficult to differentiate between supplements and drugs [5,12] and uses the former to treat various medical conditions [128,153,154]. Such practices have been observed even in the US, where manufacturers are legally obligated to include the information *“This product is not intended to diagnose, treat, cure, or prevent any disease”* [9,128] on labels, unlike in the European Union. According to various authors, knowledge about supplements among some physicians and pharmacists also leaves much to be desired [155,156]. It is worth mentioning here that in Poland, considering the popular misconception that dietary supplements are drugs, as of 1 January 2020, self-regulation of TV broadcasting companies and supplement manufacturers [157] was introduced. The new regulations have introduced several important changes for TV advertising, chief among them the rule that every advertisement is required to include the following statement on screen: *“Dietary supplement; contains ingredients which support physiological functions of the body by supplementing a typical diet; has no medicinal properties*.”

Considering the impact of dietary supplements on people’s health, it is important to remember that the results of short-term studies do not provide irrefutable data on their effectiveness and that even long-term studies, where lifestyle factors change over time and influence a person’s health, also pose a problem. When interpreting the results of research on intake of vitamin and mineral preparations, especially multivitamin–mineral supplements, it should also be remembered that there is a considerable degree of heterogeneity in supplement ingredients and amounts of nutrients because the composition of supplements is not directly regulated by law. Therefore, individual preparations may significantly differ from each other in dose and composition of ingredients, thus affecting research results. In addition, it should be noted that administering vitamin and mineral supplements to the study group does not mean that the control group did not gain appropriate levels of these ingredients from their daily diets, and thus does not mean that the control group was at risk of hypovitaminosis. Therefore, identifying the potential benefits of supplying nutrients in addition to the body’s normal requirements is difficult.

The author of this paper is aware of the limitations and issues in the cited studies and consequently is aware that this is not a systematic review. Therefore, it cannot be ruled out that some publications, possibly presenting different research results, may have been omitted. However, the author has made every effort to include as many publications as possible, including randomized controlled trials and meta-analyses, and to discuss the individual issues in the paper in a balanced way, bearing in mind the length of the paper. Because of the widely different categories of dietary supplements, the article does not address the health effects of all, e.g., probiotics or polyphenols; thus, the author does not present an exhaustive discussion of the health effects resulting from taking supplements.

## 5. Conclusions

In the absence of clinical trials conducted by the manufacturers of dietary supplements prior to their supplements being introduced to the market, being able to understand the health effects of using such preparations is an important public health issue. Most of the randomized controlled trials analyzed in this article found that vitamin and mineral supplements do not lower the risk of cardiovascular diseases and cancer, while the role of omega-3 fatty acids in the prevention of cardiological diseases is not conclusively agreed. The use of weight loss supplements is either of marginal benefit or is completely ineffective, while the side effects and the risk of adulteration with illegal substances constitute serious grounds for caution to be advised.

Coming back to the original question in the title of the article, it should be stated that dietary supplements are not recommended for everyone to be used to generally support health and reduce the risk of diseases, but rather to be used by those people with a prolonged nutrient deficiency in their diet or a previously diagnosed deficiency in the body. Therefore, more emphasis should be given to dietary changes, including the benefits of eating more fruits and vegetables, where vitamins and minerals occur naturally in combination with other nutrients that cannot be replicated in food supplements. People who intend to take supplements on their own accord should not choose preparations with the highest nutrient content because, unlike ordinary foods, excessive intake may have serious health consequences.

## Figures and Tables

**Table 1:** Characteristic of studies evaluating the effect of selected dietary supplements on cardiovascular diseases.

Authors	Study Design	Participants	Type of Dietary Supplements	Duration of the Study	Results
The ASCEND Study Collaborative Group 2018 [22]	RCT	15,480 patients with diabetes aged ≥ 40 years	Omega-3 fatty acids	Mean: 7.4 years	No effect on serious vascular events
Manson et al., 2019 [23]	RCT	25,871 patients aged ≥ 50 years	Omega-3 fatty acids	Median: 5.3 years	No effect on cardiovascular events
Bhatt et al., 2019 [24]	RCT	8179 patients with cardiovascular diseases or diabetes, median age: 64 years	Purified EPA ethyl ester (icosapent ethyl)	Median: 4.9 years	25% reduction in primary endpoints: cardiovascular death, non-fatal MI, non-fatal stroke, coronary revascularization, unstable angina; 26% reduction in secondary endpoints: cardiovascular death, non-fatal MI, non-fatal stroke
Nicholls et al., 2020 [27]	RCT	13,078 patients with high cardiovascular risk, mean age: 62.5 years	Omega-3 fatty acids	Over 3 years	No effect on major cardiovascular events
Rizos et al., 2012 [29]	Systematic review and meta-analysis, 20 RCTs	68,680 patients aged 49–70 years	Omega-3 fatty acids	Median: 2.0 years	No effect on cardiac death, MI, and stroke
Kotwal et al., 2012 [30]	Meta-analysis, 20 RCTs	62,851 patients in primary and secondary prevention settings	Omega-3 fatty acids	6 months–6 years	14% reduction in vascular death; no effect on cardiovascular events, coronary events, cerebrovascular events, arrhythmia
Kwak et al., 2012 [31]	Meta-analysis, 14 RCTs	20,485 patients with cardiovascular diseases aged 40–80 years	Omega-3 fatty acids	1.0–4.7 years	No effect on cardiovascular events, MI, congestive heart failure, and stroke
Casula et al., 2013 [32]	Meta-analysis, 11 RCTs	15,348 patients with cardiovascular diseases	Omega-3 fatty acids	1.0–3.5 years	32% reduction in cardiac death, 25% reduction in MI; no effect on stroke
Wen et al., 2014 [33]	Meta-analysis, 14 RCTs	32,656 patients with CHD	Omega-3 fatty acids	<3 months–4.6 years	12% reduction in death from cardiac causes, 14% reduction in sudden cardiac death
Abdelhamid et al., 2018 [35]	Meta-analysis, 79 RCTs	112,059 adults in primary and secondary prevention settings	Omega-3 fatty acids	12–72 months	Little or no effect on cardiovascular mortality, CHD mortality, cardiovascular events, stroke, arrhythmia
Aung et al., 2018 [34]	Meta-analysis, 10 RCTs	77,917 high-risk patients	Omega-3 fatty acids	1.0–6.2 years	No effect on CHD mortality, non-fatal MI, CHD events, major vascular events
Mazidi et al., 2019 [36]	Meta-analysis, 13 RCTs	127,447 patients	Omega-3 fatty acids	No data	9% reduction in CHD death, 5% reduction in major vascular event, 11% reduction in non-fatal MI, 5% reduction in all-cause mortality
Hu et al., 2019 [37]	Meta-analysis, 13 RCTs	127,447 patients, mean age: 64.3 years	Omega-3 fatty acids	5 years	8% reduction in MI, 8% reduction in CHD death, 5% reduction in total CHD, 7% reduction in CVD death, 3% reduction in total CVD
Casula et al., 2020 [38]	Meta-analysis, 16 RCTs	81,073 participants	Omega-3 fatty acids	≥1 year	9% reduction in cardiac mortality, 10% reduction in major adverse cardiovascular events, 17% reduction in MI; more benefits were seen in secondary prevention
Lombardi et al., 2020 [39]	Meta-analysis, 14 studies	125,763 patients	Omega-3 fatty acids	Median: 4.6 years	21% reduction in cardiac death, 29% reduction in MI, 26% reduction in coronary revascularization, 27% reduction in unstable angina, and 22% reduction in major vascular events—for high-dose omega-3 fatty acids (> 1 g per day); 49% increase in bleeding events and 35% increase in atrial fibrillation events—for high-dose omega-3 fatty acids (> 1 g per day)
Wang et al., 2009 [51]	RCT	128 obese women aged 18–50 years with hypertension or/and hyperglycemia, and/or hyperlipemia	Multivitamins and minerals	26 weeks	Significant reduction of systolic and diastolic BP
Park et al., 2011 [55]	Cohort study	182,099 patients aged 45–75 years	Multivitamins	Mean: 11 years	No effect on mortality from cardiovascular diseases
Sesso et al., 2012 [56]	RCT	14,641 males aged ≥ 50 years	Multivitamins	10.7–13.3 years	No effect on cardiovascular events, MI, stroke, cardiovascular disease mortality
Rautiainen et al., 2016 [50]	Prospective cohort study	28,157 women aged ≥ 45 years free of hypertension at baseline	Multivitamins	Mean: 11.5 years	No association with the risk of hypertension
Albert et al., 2008 [57]	RCT	5442 women with a history of cardiovascular diseases or three or more coronary risk factors aged ≥ 40 years	Folic acid, vitamin B6, vitamin B12	7.3 years	No significant effect on risk of major cardiovascular events
Galan et al., 2010 [58]	RCT	2501 patients with a history of ischemic heart disease or stroke, mean age: 60.9 years	Folic acid, vitamin B6, vitamin B12	Median: 4.7 years	No significant effect on risk of major cardiovascular events
Huo et al., 2015 [59]	RCT	20,702 patients with hypertension, without a history of stroke or myocardial infarction aged 45–75 years	Enalapril, folic acid	Median: 4.5 years	21% reduction in first stroke and 20% reduction in composite cardiovascular events
Lee et al., 2005 [69]	RCT	39,876 healthy women aged ≥ 45 years	Vitamin E	Mean: 10.1 years	No effect on MI, stroke; 24% reduction in cardiovascular death
Lonn et al., 2005 [70]	RCT	9541 patients with vascular disease or diabetes	Vitamin E	Median: 7.0 years	13% increase in heart failure
Wang et al., 2019 [71]	Mendelian randomization study	7781 participants	Vitamin E	No data	Genetically determined higher blood levels of vitamin E increases the risk of CAD and MI, increases the concentration of LDL cholesterol and triglycerides, decreases HDL cholesterol concentration
Schwingshackl et al., 2017 [13]	Meta-analysis, 49 RCTs	287,304 participants	Vitamins and minerals	1.0–11.2 years	12% reduction in cardiovascular mortality with vitamin E supplementation; no beneficial effect from other vitamin/mineral supplementation
Kim et al., 2018 [60]	Meta-analysis, 18 clinical trials and prospective cohort studies	2,019,862 participants	Multivitamins and minerals	5.0–19.1 years	No effect on cardiovascular mortality, CHD mortality, stroke mortality, stroke incidence
Li et al., 2018 [52]	Meta-analysis, 12 RCTs	23,207 patients aged 21.9–64.7 years	Multivitamins and minerals	1.0–86.4 months	No effect on risk of hypertension; significant reduction of systolic BP in subjects with hypertension
Khan et al., 2019 [61]	An umbrella review: 9 systematic review, 4 RCTs, 105 meta-analyses	992,129 participants	Multivitamins and antioxidants	No data	No effect on cardiovascular diseases outcomes
Loffredo et al., 2015 [73]	Meta-analysis, 16 RCTs	Up to 39,876 patients aged > 50 years	Antioxidants	0.5–9.4 years	18% reduction in MI with vitamin E supplementation
Vivekananthan et al., 2003 [72]	Meta-analysis, 32 RCTs	81,788 patients	Vitamin E, beta- carotene	1.4–12.0 years	No effect from supplementation of vitamin E on cardiovascular mortality, cerebrovascular events; significant increase in cardiovascular death with beta-carotene supplementation
Poorolajal et al., 2017 [53]	Meta-analysis, 23 RCTs	1213 patients with hypertension, mean age: 19–75 years	Potassium	4–52 weeks	Significant reduction of systolic and diastolic BP
Filippini et al., 2020 [54]	Meta-analysis, 32 RCTs	1764 patients mainly with hypertension aged 18–79 years	Potassium	4–15 weeks	Significant reduction of systolic and diastolic BP

Abbreviations: RCT—randomized controlled trial; MI—myocardial infarction; CHD—coronary heart disease; CAD—coronary artery disease; BP—blood pressure.

**Table 2:** Characteristic of studies evaluating the effect of selected dietary supplements on cancers.

Authors	Study Design	Participants	Type of Dietary Supplements	Duration of the Study	Results
Omenn et al., 1996 [80]	RCT	18,314 smokers	Beta-carotene and vitamin A	Mean: 4 years	28% increase in lung cancer incidence
Albanes et al., 1996 [81]	RCT	29,133 smokers aged 50–69 years	Beta-carotene, vitamin E	Median: 6.1 years	16% increase in lung cancer incidence with beta-carotene supplementation; no effect on lung cancer incidence with vitamin E supplementation.
Lee et al., 2005 [69]	RCT	39,876 healthy women aged ≥ 45 years	Vitamin E	Mean: 10.1 years	No effect on cancer incidence
Gaziano et al., 2009 [77]	RCT	14,641 men aged ≥ 50 years	Vitamin E, vitamin C	Mean: 8.0 years	No effect on cancer incidence
Klein et al., 2011 [78]	RCT	34,887 men aged ≥50 years	Vitamin E, selenium	7.0–12.0 years	17% increase in prostate cancer incidence with vitamin E supplementation; no effect on prostate cancer incidence with selenium supplementation
Park et al., 2011 [55]	Cohort study	182,099 patients aged 45–75 years	Multivitamins	Mean: 11 years	No effect on cancer incidence
Ambrosone et al., 2019 [99]	Prospective study	1134 patients with breast cancer	Antioxidants, multivitamins vitamin B12, iron,	6 months	41% increased risk of recurrence with antioxidant supplementation both before and during chemotherapy; no effect of multivitamins on survival outcomes; 2-fold decrease in the probability of disease-free survival for vitamin B12 supplementation both before and during chemotherapy; 79% higher risk of recurrence with iron supplementation both before and during chemotherapy
Narita et al., 2018 [83]	Prospective study	79,705 participants	Retinol, vitamin C, vitamin E, alfa- carotene, and beta-carotene	Mean: 5 years	26% increase in lung cancer incidence in men with higher dietary retinol intake; no associations with lung cancer incidence for vitamin C, vitamin E, alfa-carotene and beta-carotene intake
Van Gorkom et al., 2019 [96]	A systematic review, 19 trials	No data	Vitamin C	1 week–12 months	No positive effect of vitamin C supplementation on cancer patients
Pais et al., 2013 [85]	Meta-analysis, 20 RCTs	268,590 participants	Antioxidants	No data	No effect on colorectal cancer incidence with antioxidant supplementation
Park et al., 2017 [79]	Meta-analysis, 14 RCTs	147,383 participants	Antioxidants	1.0–13.0 years	No effect on bladder cancer incidence with antioxidant supplementation
Cortés-Jofré et al., 2020 [84]	Meta-analysis, 12 RCTs	733–212,314 participants 35–84 years	Antioxidants	2.0–12.0 years	No beneficial effect on lung cancer incidence for combination of vitamins A, C, E + selenium + zinc supplementation; 84% increase in lung cancer incidence in women with vitamin C supplementation; 10% increase in lung cancer incidence in smokers and people with history of asbestos exposure with vitamin A supplementation
Bjelakovic et al., 2014 [91]	Meta-analysis, 18 RCTs	50,623 participants	Vitamin D	Mean: 6 years	No effect on cancer incidence; 12% reduction in cancer death.
Goulao et al., 2018 [89]	Meta-analysis, 30 RCTs	18,808 participants	Vitamin D	Median: 1,0–6.2 years	No effect on cancer incidence and mortality
Keum et al., 2019 [90]	Meta-analysis, 5–10 RCTs	6537 cases	Vitamin D	3–10 years	No effect on cancer incidence; 13% reduction in cancer death

**Table 3:** Characteristic of studies evaluating the effect of selected dietary supplements on weight loss.

Authors	Study Design	Participants(*n*)	Type of Dietary Supplements	Duration of the Study	Results
Onakpoya et al., 2013 [110]	Meta-analysis, 20 RCTs	1038	Chromium	8–26 weeks	0.5 kg more weight loss as compared with placebo
Tsang et al., 2019 [111]	Meta-analysis, 21 trials	1316	Chromium	≤12 weeks	0.75 kg more weight loss as compared with placebo
Moraru et al., 2018 [113]	Meta-analysis, 14 RCTs	1101	Chitosan	4–52 weeks	1.01 kg more weight loss as compared with placebo
Huang et al., 2020 [115]	Meta-analysis, 15 RCTs	1130	Chitosan	≥12 weeks	0.89 kg more weight loss, 0.39 kg/m^2^ more BMI loss, and 0.69% more body fat loss as compared with placebo
Baladia et al., 2014 [116]	Meta-analysis, 5 RCTs	260	Green tea, green tea extract	12 weeks	No effect on body weight
Lin et al., 2020 [117]	Meta-analysis, 22 RCTs	2357	Green tea extract	4–14 weeks	1.78 kg more weight loss and 0.65 kg/m^2^ more BMI loss as compared with placebo
Onakpoya et al., 2011 [120]	Meta-analysis, 12 RCTs	706	hydroxycitric acid from *Garcinia cambogia*	2–12 weeks	0.88 kg more weight loss as compared with placebo
Golzarand et al., 2020 [121]	Meta-analysis, 8 RCTs	530	*Garcinia cambogia*	8–12 weeks	1.34 kg more weight loss, 0.99 kg/m^2^ more BMI loss, and 0.42% more body fat loss, 4.16 cm more loss of waist circumference as compared with placebo
Stohs 2017 [123]	Review 30 trials	600	*Citrus aurantium* extract	No data	No proven effect on body weight
